# A novel systems pharmacology model for herbal medicine injection: a case using reduning injection

**DOI:** 10.1186/1472-6882-14-430

**Published:** 2014-11-04

**Authors:** Haixing Yang, Wenjuan Zhang, Chao Huang, Wei Zhou, Yao Yao, Zhenzhong Wang, Yan Li, Wei Xiao, Yonghua Wang

**Affiliations:** Center of Bioinformatics, Northwest A & F University, Yangling, Shaanxi 712100 China; College of Life Sciences, Northwest A & F University, Yangling, Shaanxi 712100 China; College of Life Science, Northwest University, Xi’an, Shaanxi 710000 China; Jiangsu Kanion Parmaceutical Co.LtD, Lianyungang, Jiangsu 222002 China; State Key Laboratory of New-tech for Chinese Medicine Pharmaceutical Process, Lianyungang, Jiangsu 222002 China; Lab of Pharmaceutical Resource Discovery, Dalian Institute of Chemical Physics, Chinese Academy of Sciences, Dalian, Liaoning 116023 China

**Keywords:** Systems pharmacology, Reduning injection, Polypharmacology, Mechanism of action

## Abstract

**Background:**

Compared with the traditional oral administration form, injection administration is basically superior in terms of both biological availability and therapeutic effects. However, few researches have focused on the traditional Chinese medicinal injection due to the complicated constituents and the intricate mechanism of action.

**Methods:**

In the present work, a novel systems pharmacology model, integrating ADME (absorption, distribution, metabolism, and excretion) filtering such as half-life evaluation, network targeting, pathway and systems analyses, is specifically developed for the identification of active compounds and the study of the mechanism of action of TCM injection, which is exemplified by Reduning injection confronting the influenza.

**Results:**

The ADME filter successfully identifies 35 bioactive compounds (31 molecules and 4 metabolites) from the Reduning injection. The systems analysis and experimental validation further reveal a new way of confronting influenza disease of this injection: 1) stimulating the immunomodulatory agents for immune response activation, and 2) regulating the inflammatory agents for anti-inflammation.

**Conclusions:**

The novel systems pharmacology method used in this study has the potential to advance the understanding of the molecular mechanisms of action of multicomponent herbal injections, and provide clues to discovering more effective drugs against complex diseases.

**Electronic supplementary material:**

The online version of this article (doi:10.1186/1472-6882-14-430) contains supplementary material, which is available to authorized users.

## Background

Traditional Chinese Medicine (TCM), a typical ethnomedicine derived from the practice of ancient Chinese herbal medicine through several thousand years of empirical testing and refinement, has been successfully used in taming various kinds of diseases [1]. The most common practice in TCM is the utilization of herbal combinations called formulae, which are capable of systematically controlling various complex diseases determined by synergistic effects among different herbs. As demonstrated in the clinical trials, the injection, also featured as “multiple components, multiple targets and complex diseases”, is normally superior to the traditional oral administration form in the matter of both biological availability and therapeutic effects [2]. However, even though mixtures in herbal injection have been investigated through observation and experience over years, the mechanism of action is still unknown due to the insufficient modern scientific research [3]. Thus modern and technologic approaches are urgently needed for the study of TCM injections.

Fortunately, the advent of -omics technologies rapidly integrate the entirety of the human complement (such as genomics and metabonomics) to propose a new way of study TCM in the form of systems biology [4]. To conduct a systems-level analysis, a comprehensive analysis of the dynamic interactions between drug(s) and a biological system is required. Hence, bridging systems biology and pharmacokinetics-pharmacodynamics (PK/PD) has led to the emergence of systems pharmacology [5]. The term systems pharmacology describes a field of study that applies the systems biology and PK/PD to provide a quantitative frame-work for understanding the dynamic interplay among variables of complex biological systems through iteration between computational and/or mathematical modelling and experimentation [5, 6]. The application of systems pharmacology can impact across a wide range of drug research and development stages. In fact, systems pharmacology has been successfully applied to TCM for screening bioactive drug ingredients [7], predicting drug targets [8], understanding therapeutic mechanisms [9–11], revealing rules of drug combination [12], screening synergistic drug combinations [13], and so forth. However, an effective method which is specifically developed for the study of herbal injection is still lacking at the present time. Therefore, in this work, a novel systems pharmacology-based strategy is presented for the study of herbal injections. It is exemplified by a widely used TCM injection Reduning, which mainly treats influenza diseases including virus infection, fever, respiratory disease, inflammation, etc. [2, 14].

The whole system includes four steps: (1) components collection in Reduning injection; (2) ADME filter building and screening; (3) systems analysis for the action mechanisms of Reduning; and (4) the experimental verification. This work pays a great deal of attention to the ADME prediction in injection, encompassing results of permeability, cell uptake, blood–brain penetration, protein transporting and binding, metabolism of bioactive substances, and drug excretion. Furthermore, the application of this systems pharmacology model may reveal the power of the combined approach for screening bioactive compounds, predicting the mechanisms of action, and understanding the multicomponent therapeutic efficacy.

## Methods

### Chemical database for reduning injection

Reduning injection consists of three herbs including *Artemisiae annuae* L. (genus *Artemisia*, Asteraceae), *Gardenia jasminoides J.Ellis* (genus *Gardenia*, Rubiaceae) and *Lonicera japonica Thunb.* (genus *Lonicera*, Caprifoliaceae). In our previous work, 69 chemical constituents have been successfully isolated and identified as seen in Additional file 1: Table S1, including 15 iridoid compounds, 12 lignan compounds, 11 phenolic acid compounds, 10 flavonoids compounds, 10 caffeoylquinic acid derivatives, 5 sesquiterpenoid compounds, 3 coumarin compounds, etc. [15]. In addition, since the glycosyl groups of 17 compounds may be deglycosylated by the rule of glycosidase hydrolysis reaction, their aglycons labeled by _sg are also added, thus a total of 86 compounds are included in the present analysis (Additional file 1: Table S1).

### Generation of ADME filters

**Figure 1:**
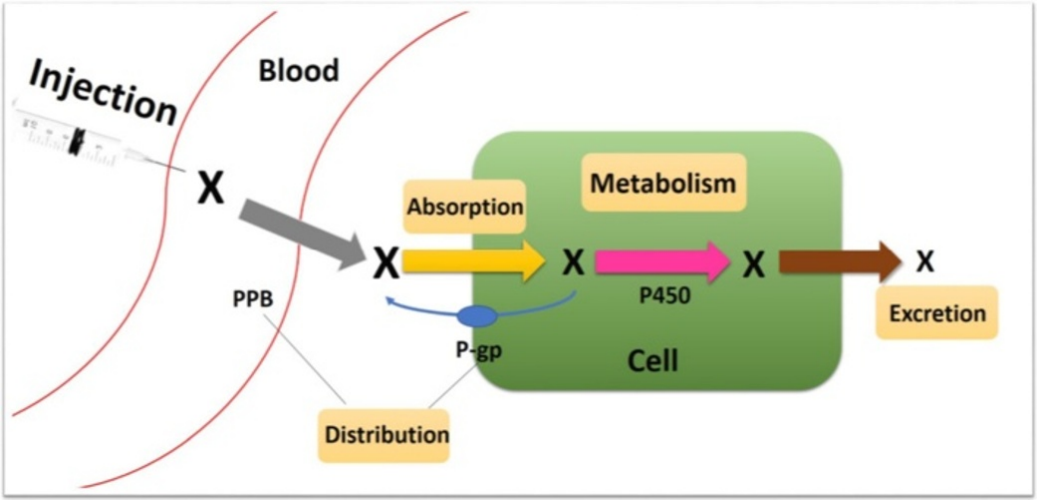
**The integrative ADME filtering model.** The model includes the key ADME process such as membrane penetrating, cell uptake, protein transporting and binding, metabolism of bioactive substances, and drug excretion. Abbreviations: PPB, Plasma-protein binding; P-gp, P-glycoprotein; P450, cytochrome P450.

To ensure a continuous pipeline of new drugs with reasonable PK, one pivotal activity is the evaluation of ADME parameters for a given compound. For this purpose, a novel systematic ADME prediction model is developed as seen in Figure 1.

#### Lipophilicity

It is one of the key physicochemical parameters linking membrane permeability with the route of clearance [16]. The partition coefficient P (log P) is calculated by ALOGPS 2.1 software [17] in this work. The threshold value of log P is set to 5 according to the Lipinski’s rule of five [18].

#### Aqueous solubility

Log S, a measure of aqueous solubility, is another crucial property of drugs [19], which is also calculated using ALOGPS 2.1 software [17] and the threshold value here is −5< log S< −1 [20].

#### P-glycoprotein (P-gp)-medicated interaction

P-gp functions as a biological barrier by active efflux of a wide variety of structurally and chemically unrelated compounds from cells [21]. In order to classify the potential P-gp substrates and inhibitors, our previous model based on the Kohonen self-organizing map artificial neural network is applied here [22].

#### Plasma-protein binding (PPB) evaluation

Binding ability of a drug to human serum albumin is one of the most factors that influence drug distribution [23]. In this work, the model which integrates support vector machine (SVM) prediction and the molecular docking method is used to predict whether a compound can bind to albumin [24].

#### Metabolism-related drug-drug interactions

Cytochromes P450 play a crucial role in metabolism [25], and the potent inhibitors of P450 can lead to undesirable drug-drug interactions when co-administered with other drugs [26]. Thus, a model which built from experimental high-throughput data using SVMs and molecular signatures is used to predict whether the compound is a CYP inhibitor [27]. Here, three most important P450 (CYP) 3A4, 2D6, and 2C9 which account for more than 80% drug metabolism [28] are taken into consideration in this work.

#### Half-life prediction

Half-life (t_1/2_), which is defined as “the time taken for the amount of compound in the body to fall by half”, is arguably the most important property as it dictates for the timescale over which the compound may elicit therapeutic effects [29]. In this work, a novel *in silico* model (PreDHL) is generated to predict long or short half-life of drugs by using the C-partial least square (C-PLS) algorithm [30–32]. The building mainly includes the following three steps:

1. *Data sets collection.* A total of 169 drugs (injection formulation) with their half-life values, DrugBank ID, chemical name, CAS number were collected from Drugbank database (http://www.drugbank.ca/) [33] (Additional file 2: Table S2). 4 hour of half-life value was regarded as the judging boundary for long half-life (half-life value ≥4 h) and short half-life (half-life value<4 h). This dataset was then split into two subsets, i.e., a training set (n=126) used to build the model and an independent test set (n=43) to validate the accuracy of the model;

(2) *Descriptor calculation and selection.* Molecular descriptors were firstly calculated to construct the model, 1664 chemical descriptors were calculated using DRAGON 6 program (http://www.talete.mi.it/index.htm), which is a useful tool to evaluate the molecular structure–activity or structure–property relationships [34]. Then 43 objective features were selected based on forward stepwise algorithm. Finally, principal component analysis (PCAs) was employed to reduce the dimensionality of the objective features and eventually 8 (Additional file 2: Table S2) of them were obtained and further applied for C-PLS modeling process. C-PLS was carried out by the TANAGRA (version 1.4.38, http://eric.univ-lyon2.fr/~ricco/tanagra/en/tanagra.html);
(3) *Model performance.* With the purpose of deriving reliable *in silico* models, both internal and external validation methods were applied. For the internal validation, the half-life prediction model was evaluated and verified with leave-one-out (LOO) methodology. Meanwhile, external validation was performed by using the test sets for all models. The prediction performance in the classification system was evaluated by the parameters: overall, short half-life and long half-life accuracies. As a result, the derived model shows impressive performance of prediction for half-life. For internal validation, the overall accuracy, long half-life accuracy, and short half-life prediction accuracy are 85.21%, 84.81% and 85.56% respectively; for external validation, the overall accuracy is 86.05%, the long half-life accuracy is 85.00%, and the short half-life accuracy is 86.96%.

### Tanimoto similarity (TS)

Drug-like compounds are those which ‘contain functional groups and/or have physical properties consistent with the majority of known drugs’ [35]. Hence, the Tanimoto coefficient [36] is used to remove compounds which are deemed to be chemically unsuitable for drugs, and the TS index is introduced to describe how herbal compounds are comparable to known drugs in Drugbank database. The TS index is defined as following:


where, x and y represent the structural feature vectors of two compounds, respectively. In this work, the TS ≥0.18 (average value of drugs in Drugbank) is defined to select drug-like compounds.

### Drug targeting

Comprehensively determining compound-target interaction profiles is a critical step for elucidating the mechanisms of drug action [37]. To predict the target profiles of active herbal compounds accurately, an overall drug targeting strategy integrating our *in silico* prediction model, chemogenomics method and publicly database interrogation strategy is developed as following: (1) Our *in silico* prediction model efficiently integrates the chemical, genomic, and pharmacological information for drug targeting on a large scale, which based on two powerful methods: Random Forest (RF) and SVM [38]. In cases where drug targets are identified, proteins with an output expectation value: SVM >0.7 or RF >0.8 are listed as potential targets; (2) SEA search tool (SEArch, http://sea.bkslab.org/), the online search tool for the Similarity Ensemble Approach [39], where relates proteins based on the chemical similarity of their ligands. The final score is expressed as an expectation value (E-value), that is, the structural similarity of each drug to each target’s ligand set; and (3) STITCH 4.0 (Search Tool for Interacting Chemicals, http://stitch.embl.de/), a combined data repository that captures the publicly available knowledge on chemical-protein interactions derived from experiments, expert-curated databases and literature by means of text mining [40].

Furthermore, the final obtained target proteins were applied as baits to fish their related diseases and pathways. The target-disease relationships were retrieved from the TTD database (Therapeutic Target Database, http://bidd.nus.edu.sg/group/cjttd/), and the US National Library’s Medical Subject Headings (http://www.nlm.nih.gov/mesh), where the diseases can be classified into different groups. The target-pathway relationships were obtained from the KEGG database (Kyoto Encyclopedia of Genes and Genomes, http://www.genome.jp/kegg/).

### Network generation and topological analysis

In systems pharmacology, network formed by nodes and edges (connections between nodes), is a mathematical, computable and quantifiable description of various relationships under the complex biological systems [41]. Network parameters, such as degree and betweenness - the basic network topological properties - can be utilized to describe the characterization of different drug treatments from a network perspective [42]. The degree of a node is the number of edges associated to it, and the betweenness of a node is the number of shortest communication paths between different pairs of nodes. The nodes with high centrality (degree and betweenness) can be considered the key nodes in a network [43]. The topological properties of these networks were analyzed using Network Analysis plugin and CentiScaPe 1.2 of Cytoscape [44].

### Measurement of nitrite concentration for experimental validation

RAW 264.7 mouse macrophage-like cells were obtained from the Shanghai Institute of Cell Biology, Chinese Academy of Sciences (Shanghai, China) and maintained at 37°C in a 5% CO_2_. The medium used for routine subculture was DMEM (Gibco, USA) supplemented with 10% FBS (HyClone, USA). For experiments, the cells were plated at a density of 4 × 10^5^ cells/ml into 96-well plates containing 100 ul medium. After 24 h incubation, the culture medium was replaced with fresh medium and the cells were treated with M38, M56 and M57 respectively at different concentrations for 2 h, followed by stimulation with LPS (Sigma, USA) 100 ng/ml for an additional 20 h. Then the level of NO production was monitored by Griess method and according to the indication on the NO assay kit (Beyotime Biotechnology, China).

## Results and discussion

### Druglikeness filtering

The high failure rate (50%) of drug development in clinical trials is partly due to the poor PK properties of drug candidates [45]. Moreover, an effective method which is specifically developed for the study of herbal injection is still lacking at the present time. Thus, establishing the methods for the effective screening of compounds with optimal ADME properties is of obvious necessity. As a matter of fact, a successful drug is the combination of biological activity and drug-like properties; in addition, property screening allows us to optimize ADME properties in parallel with drug-like properties [46]. Hence, the critical PK properties of an injection administered drug can be illuminated by examining those parameters directly correlated with ADME processes: Log P, Log S, P-gp, PPB, P450 and half-life, and the TS index.

Compounds that successfully meet the ADME properties criteria include: overcoming 62.5% (5/8) of the ADME barriers and the appropriate TS (≥0.18) are nominated as candidates for formal development. Consequently, out of the obtained 86 compounds, 31 molecules with 4 metabolites have favorable PK properties are shown in Table 1. Among them, neochlorogenic acid (M56), which is one of the most principal active compounds of *Lonicera japonica Thunb.*, has been proved with good pharmacological effects as predicted in the current study [47]. Two well-known flavonoids, quercetin (M30) and luteolin (M34) have been proven to overcome various ADME barriers through inhibition of P-gp [48] and cytochrome P450 [49–51]. Moreover, geniposide (M4), one of the major iridoid glycosides in *Gardenia jasminoides J.Ellis* [52], is the potential inhibitor of cytochrome P450 [53] and substrate of P-gp [54].

**Table 1:** **Pharmacokinetic property predictions for the 35 molecules and their network parameters**

ID	Compound	logS	logP	2C9	2D6	3A4	PPB	P-gp	Y(t1/2)	TS	Betweenness	Degree	Structure
M1	L-phenylalaninosecologanin B	+	+	+	U.C.	+	+	+	-	0.34	1.60E-02	7	
M2	L-phenylalaninosecologanin C	+	+	+	U.C.	+	+	-	-	0.84	9.46E-03	5	
M3	secoxyloganin	+	+	+	U.C.	+	+	-	-	0.39	1.32E-02	6	
M4	geniposide	+	+	+	U.C.	+	+	-	-	0.44	7.08E-02	11	
M8	5H,8H-pyrano[4,3-d]thiazolo[3,2-a]pyridine-3-carboxylic acid	+	+	+	U.C.	+	+	-	-	0.79	0	0	
M9	vogeloside	+	+	+	U.C.	+	+	-	-	0.46	1.00E-05	2	
M10	7-epi-vogeloside	+	+	+	U.C.	+	+	-	-	0.46	1.00E + 00	2	
M13	7,8,11-trihydroxy-1-hydroperoxy-4-guaien-3-one	+	+	U.C.	U.C.	+	+	+	-	0.18	2.74E-02	4	
M17	3, 3′, 5-trimethoxy-4′, 7-epoxy-8, 5′-neolignan-4, 9, 9′-triol	+	+	+	U.C.	+	-	-	+	0.47	0	0	
M19	5-benzofurancarboxylic acid, 2,3-dihydro-2-(4-hydroxy-3-methoxyphenyl)-3-(hydroxymethyl)-7-methoxy	+	+	+	U.C.	+	-	+	-	0.36	6.70E-02	26	
M20	5-benzofurancarboxylic acid	+	+	+	U.C.	+	-	+	+	0.44	6.93E-02	27	
M21	threo-1-(4-hydroxy-3-methoxyphenyl)-2-[2-hydroxy-4-(3-hydroxypropyl)phenoxy]-1, 3-propanediol	+	+	+	U.C.	+	+	-	-	0.35	8.84E-02	23	
M30	Quercetin	+	+	+	-	-	-	+	-	0.28	1.75E-01	39	
M31	Rutin	+	+	+	U.C.	+	+	-	+	0.68	6.51E-02	20	
M33	luteolin 7-O-β-D-glucoside	+	+	+	+	+	-	-	+	0.78	2.65E-02	13	
M34	Luteolin	+	+	+	-	-	-	+	+	0.25	1.20E-01	35	
M38	3,5-di-O-caffeoylquinic acid	+	+	+	+	+	-	-	-	0.68	1.58E-02	8	
M39	3,5-di-O-caffeoylquinic methyl ester	+	+	+	+	+	-	-	-	0.68	1.45E-02	7	
M41	4,5-di-O-caffeoylquinic methyl ester	+	+	+	U.C.	+	-	-	-	0.69	1.01E-02	10	
M54	(2E,6S)-8-[α-L-arabinopyranosyl-(1″-6′)-β-D-glucopyranosyloxy]-2,6-dimethylct-2-eno-1,2″-lactone	+	+	+	U.C.	+	+	-	-	0.78	1.10E-04	2	
M55	5-O-caffeoylquinic methyl ester	+	+	+	+	+	+	+	-	0.36	2.25E-02	16	
M56	5-O-caffeoylquinic acid	+	+	+	+	+	-	+	-	0.33	4.55E-03	7	
M57	4-O-caffeoylquinic acid	+	+	+	U.C.	+	-	+	-	0.33	1.74E-02	11	
M58	4-O-caffeoylquinic methyl ester	+	+	+	U.C.	+	-	+	-	0.36	1.96E-02	12	
M61	geniposidic acid	+	+	+	U.C.	+	+	-	-	0.41	1.28E-02	5	
M62	genipin-1-β-D-gentiobioside	+	+	+	U.C.	+	+	-	-	0.83	2.73E-02	4	
M63	6′′-O-trans-p-coumaroylgenipin gentiobioside	+	+	+	U.C.	+	+	-	-	0.45	0	0	
M64	6′′-O-trans-p-feruloylgenipin gentiobioside	+	+	+	U.C.	+	+	-	-	0.39	0	0	
M65	6′′-O-trans-sinapoylgenipin gentiobioside	+	+	+	U.C.	+	+	-	-	0.34	0	0	
M66	Jasmigeniposide A	+	+	U.C.	U.C.	+	+	-	+	0.40	1.00E-05	2	
M69	2′-O-trans-caffeoylgardoside	+	+	+	U.C.	+	+	-	-	0.79	0	0	
M1_sg	L-phenylalaninosecologanin B_qt	+	+	+	U.C.	+	+	+	-	0.34	2.36E-01	42	
M2_sg	L-phenylalaninosecologanin C_qt	+	+	+	U.C.	+	+	-	-	0.37	2.63E-01	45	
M8_sg	5H,8H-pyrano[4,3-d]thiazolo[3,2-a]pyridine-3-carboxylic acid_qt	+	+	U.C.	U.C.	+	+	+	-	0.23	1.37E-02	2	
M66_sg	Jasmigeniposide A_qt	+	+	U.C.	U.C.	+	+	-	+	0.74	3.60E-04	2	

U.C.: uncertain; +: the compound can overcome the barriers; −: the compound cannot overcome the barriers.

It should be emphasized that any single screening paradigm does not fit all discovery projects [55]. In this study, the most common ADME properties are simultaneously visualized in drug discovery research, elucidating the basic and complex trends for multiple properties across various functional groups [56]. The incorporation of the key ADME properties is firstly used as profiling filter for sieving out the most promising molecules for the traditional Chinese herbs, leading to the foundation of a systematic approach for compound selection and later stage experimentation in TCM.

### Drug-target network

**Figure 2:**
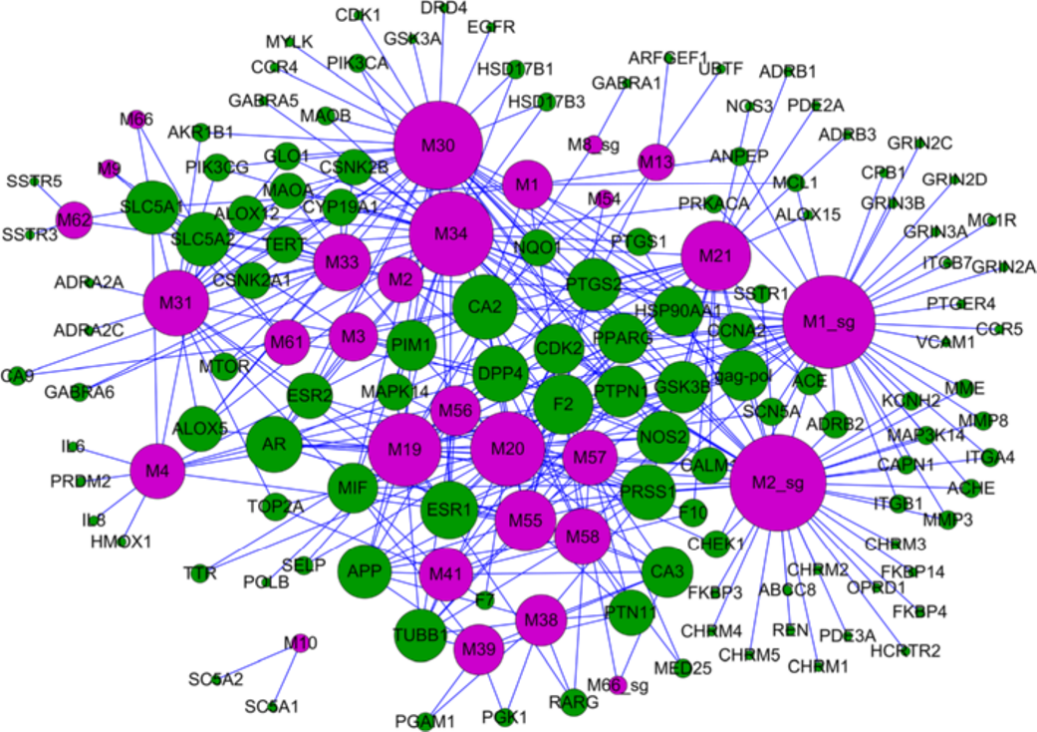
**The drug-target network.** A drug node (circle, purple) and a target node (circle, green) are connected to each other by blue edge if the protein is the target of the drug. The size of one node is proportional to its degree.

The drug-target network is defined as a physical bipartite interaction network where the color-coded nodes denote herbal drugs (purple) and their target proteins (green), and an edge links a drug node to a protein node if the protein is the target of the drug (Figure 2). It consists of 395 drug-target interactions connecting the 29 drugs to 121 targets, resulting in an average number of target proteins per drug of 13.6.

The topology of the drug-target network can be used to construct a kernel from the interaction profiles as follows: (1) In the drug-target network, as seen in Figure 2, it is apparent that most drugs show relevant polypharmacology, i.e., a drug binds to more than one target [57]. One drug binding to multiple targets may contribute to the overall effectiveness of the treatment, thereby giving rise to the therapeutic polypharmacology. The potential polypharmacological effects of all active compounds in this work are involved in the modulation of multiple targets, such as the two highly connected nodes M30 (quercetin, degree=39, betweenness=0.17509) and M34 (luteolin, degree=35, betweenness=0.11984). For example, quercetin is one of the large hubs corresponding to different target clusters. It has high affinities with Glycogen synthase kinase-3 beta (GSK3B), Nitric oxide synthase, inducible (NOS2), Prostaglandin G/H synthase 1/2 (PTGS1/2) and so forth, which induce a highly complex pharmacological profile including anti-inflammatory, antioxidant, etc. [58]. (2) It is also apparent that most of the target proteins (61.9%) are cross-linked together in this network. Hence, enhancing pharmacological synergies may be existed among active compounds due to the fact that two drugs directed at a similar receptor target or physiological system lead to pharmacodynamic synergy [59]. For example, Trypsin-1 (PRSS1, degree=11, betweenness=0.03235), a well-known proteolytic enzyme involved in various pathological processes including inflammation, abnormal blood coagulation, tumor invasion and atherosclerosis [60], possesses the large number of connections with caffeoylquinic acid derivatives like 3,5-di-O-caffeoylquinic acid (M38), 4,5-di-O-caffeoylquinic methyl ester (M41) and 5-O-caffeoylquinic methyl ester (M55). In conclusion, the drug-target network analysis can provide insights into the drug and target interation such as target binding, therapeutic polypharmacology and herbal synergism.

### Target-disease network

**Figure 3:**
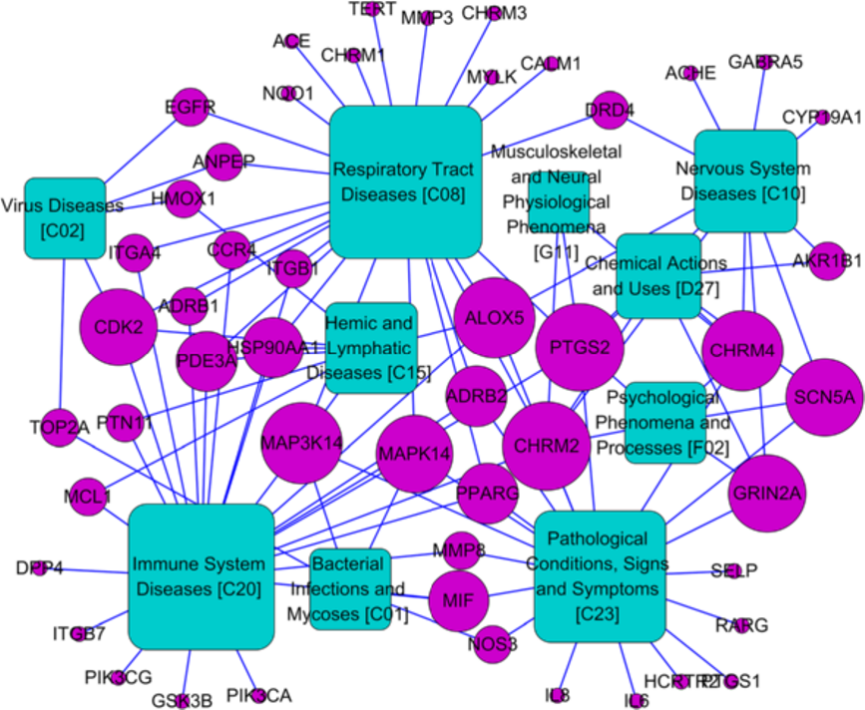
**The target-disease network.** A target node (circle, purple) and a disease node (square, green) are connected to each other by grey edge if the target is involved in the related pharmacological process. The size of one node is proportional to its degree.

Influenza is often viewed as a complex disease characterized in its full form by the sudden onset of high fever, then is the inflammation of the upper respiratory tree and trachea with coryza, cough and headache [61]. We retrieved 49 potential targets out of the predicted targets associated with influenza related diseases, and according to the US National Library’s Medical Subject Headings, these diseases were classified into 10 groups, like pathological conditions, signs and symptoms, respiratory tract diseases and immune system diseases, virus diseases, bacterial infections, and so forth. Accordingly, we constructed a target-disease network resulted in 110 target-disease interactions connecting 49 targets to 10 diseases (Figure 3), and about half of the targets (27/49) relate to multiple diseases.

Further analysis of the target-disease network shows that Reduning injection probably protects human against influenza mainly in three ways: treating respiratory tract diseases (degree=25), enhancing innate and adaptive defenses (degree=23), and treating inflammation (pathological conditions, signs and symptoms, degree=19). The overlapping targets among these three diseases (as seen in Figure 3) indicate that different diseases share common pathological changes and could be cured by a common herbal combination [62]. For example, Arachidonate 5-lipoxygenase (ALOX5) is one of the key enzymes in the formation of proinflammatory eicosanoids from arachidonic acid [63], which transforms essential fatty acids into leukotrienes (like leukotriene B4, C4, D4 and E4) [64]. In this network, it is a current target for pharmaceutical intervention against various diseases and has high affinities with compounds M30, M34, etc. Moreover, Reduning injection might also limit the virus infection by targeting the viral protein directly, like DNA topoisomerase 2-alpha (TOP2A), which inhibits the replication of virus [65].

Even though the definition of the complex mechanism underlying diseases is challenging, target interactions and their roles in understanding diseases can be characterized by the bio-molecular network model. This work gives a systems-level perspective for the further understanding of therapeutic polypharmacology at the molecular level, i.e., treating complex diseases by targeting multiple targets with more than one drug in the herbal combination [57].

### Target-pathway network and systems analysis

**Figure 4:**
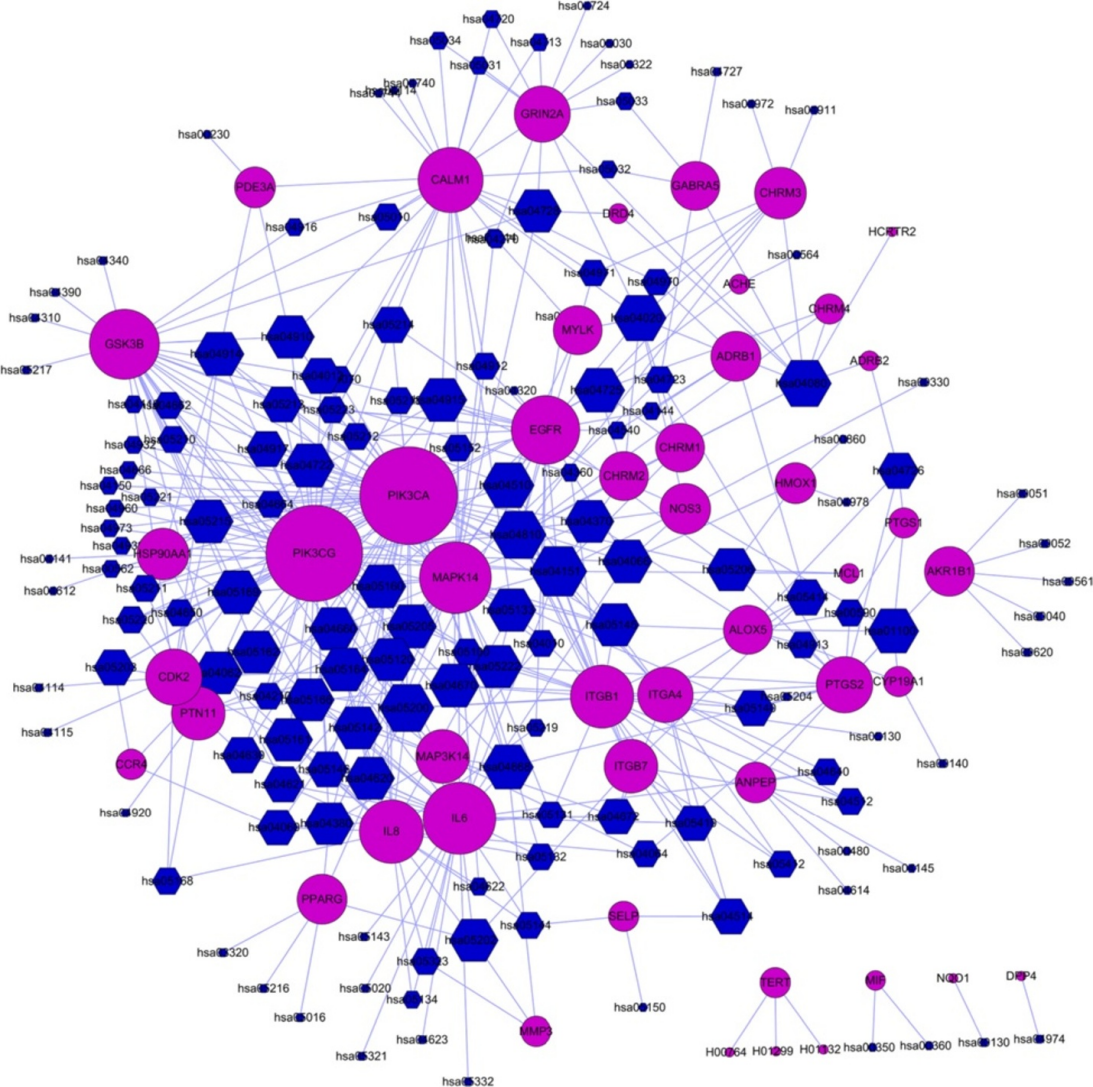
**The target-pathway network.** A target node (circle, purple) and a pathway node (hexagon, blue) are connected to each other by blue edge if the target is mapped on the pathway. The size of one node is proportional to its degree.

**Figure 5:**
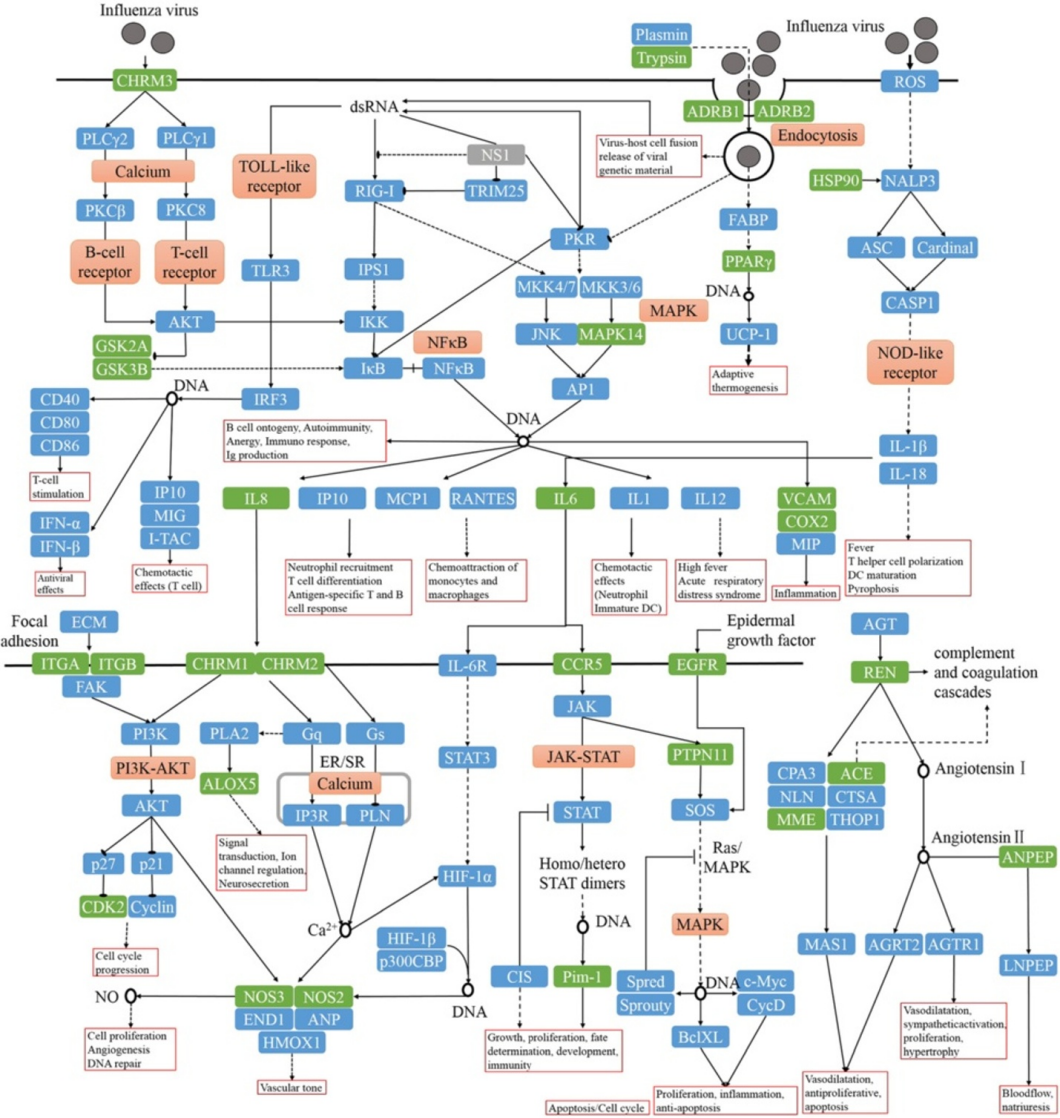
**Distribution of target proteins on the compressed ‘influenza-related pathway’.** Pathways are marked in pink and our putative drug targets are marked in green. Arrows indicate activation, T-arrows indicated inhibition, and segments indicate actions that can either be activatory or inhibitory on the specific targets.

A target-pathway network for 48 putative drug targets of Reduning and 154 KEGG pathways are illustrated in Figure 4. As can be observed, most of the target proteins (42/48) appear in multiple pathways indicating that these targets may intercede the interactions and cross-talk between different pathways. Similarly, major pathways (105/154) are also modulated through multiple target proteins, and many of them have been reported as suitable target pathways for influenza therapies, such as the PI3K-Akt signaling pathway (hsa04151) [66], Neuroactive ligand-receptor interaction (hsa04080), Calcium signaling pathway (hsa04020), and Toll-like receptor signaling pathway (hsa04620) [65].To better elaborate the molecular mechanism of Reduning injection, an integrated ‘influenza-related pathway’ is compiled based on the following steps: (1) 48 putative drug targets are used as a query to fish out their partners, and the pathways are expanded step by step; (2) closely connected proteins are grouped together; and (3) the intermediate interacting partners are removed to clearly display the underlying mechanism of Reduning. As shown in Figure 5, 48 targets can be organized into the following pathways indicating that multicomponent herbal medicines mainly control infectious diseases by modulating inflammatory and immune responses.

Influenza infection can be recognized by the immune system in multiple ways as described in Figure 5, including Toll-like receptor signaling pathway, NOD-like receptor signaling pathway, T-cell receptor signaling pathway and so forth. Host innate immune system initiates a wide range of defense mechanisms which may contribute to the development of inflammation by the establishment of a network of cytokines, chemokines and prostanoids [67]. Taking the NOD-like receptors (NLRs) as an example, which are a specialized group of intracellular receptors accompanying with a primary role in host defense against invading pathogens [67, 68]. However, in addition to their important functions in the host innate immune system, they are also involved in the pathogenesis of a variety of human inflammatory diseases due to their ability to regulate nuclear factor-kappa B (NF-κB) signaling, interleukin-1-beta (IL-1β) production [68]. Furthermore, IL-1β induces the expression of hundreds of genes, including cytokines (IL-6 and TNF-α), and pro-inflammatory mediators (iNOS, COX2, PLA2) [69]. Another example is the TOLL-like receptors (TLRs), they also have a crucial role in innate regulating immunity [70] and regulating inflammatory response by the production of inflammatory cytokines such as IL-1β, TNF-α and IL-6 [71]. The best characterized regulator of TLR signaling is controlled by the expression of many genes involved in the inflammatory response of NF-κB transcription factor [70].

Hence, multicomponent herbal medicine probably control the infectious disease mainly based on two ways: the stimulation of the immunomodulatory agents (such as GSK3B, MAPK14, PPARγ) will probably help enhancing innate and adaptive immune, and meanwhile, the regulation of the inflammatory cytokines and pro-inflammatory mediators (like IL-6, IL-8, TNF-α, COX2) by herbal ingredients will probably help diminishing inflammation. In recent years, the continuing emergence of drug resistance, vaccines and antiviral agents will fail to meet global needs for the influenza, therefore our conclusion may provide the new way of confronting the influenza for researchers and scientists. Moreover, several studies have suggested the crucial role of host response in severe influenza virus infection [72] and the importance of inflammasomes in innate immune responses [73]. Hence, the influenza virus recognition through immunomodulatory and anti-inflammatory agents should provide clues to making more effective therapeutics agents against influenza.

### Effects of M38, M56and M57 on LPS-induced NO production in RAW264.7 cells

**Figure 6:**
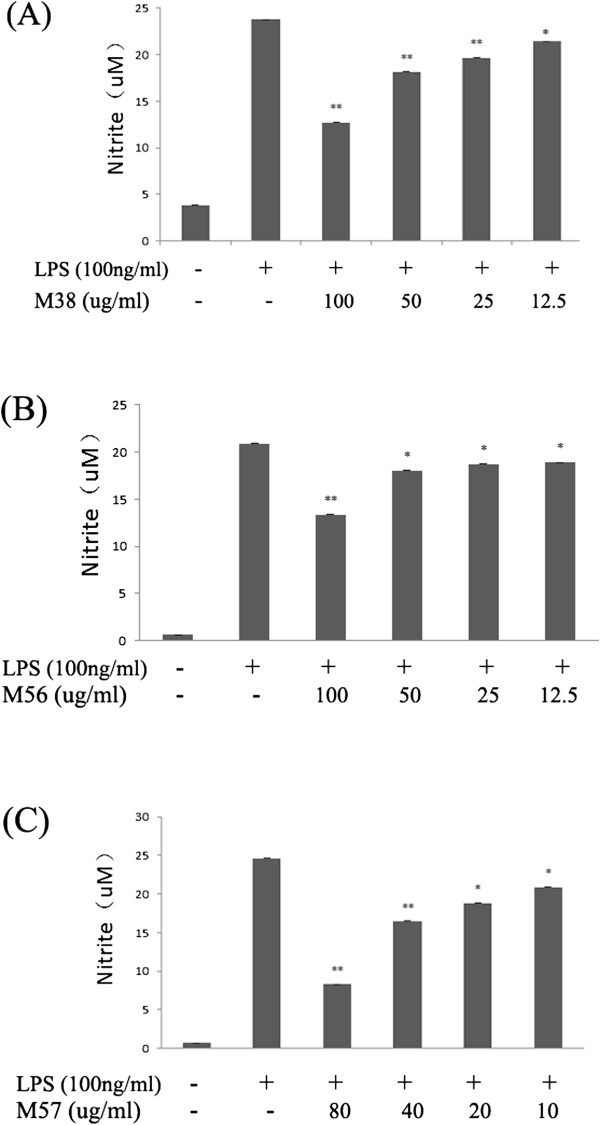
**Effect of different treatment upon LPS-induced NO release from RAW264.7 cells.** The cells were pretreated with various concentrations of M38 **(A)**, M57 **(B)** and M56 **(C)** for 2 h and then incubated with LPS (100 ng/ml) or LPS only for 20 h. Values are the mean ± SD for n= 3, *p< 0.05 and **p< 0.01.

Macrophages are considered to play a crucial role in the inflammation process [74]. Activated macrophages can generate various pro-inflammatory mediators such as TNF-α, IL-1, IL-6, NO and PGE2 [75]. Meanwhile, NO may attend almost all stages in the development of inflammation, and it can be over-produced endogenously by inducible nitric oxide synthases (iNOS) which could be induced in response of pro-inflammatory cytokines and LPS [76]. It also has been shown that the NF-κB signal pathway is crucial in the activation of immune cells by up-regulating the highly expression of many cytotoxic factors including iNOS and other pro-inflammatory cytokines [77, 78]. Therefore, in order to supply a preliminary evidence to testify the targets which we have predicted in our paper, 3 typical compounds (M38, M56 and M57) from active candidates were selected and the NO production was taken as a measuring index in our validation experiment. Then the results were expressed as mean ± SD of three independent experiments in Figure 6. The statistical significance of differences among groups were assessed using one-way analysis of variance (ANOVA) followed by the Student’s t-test. P< 0.05 was considered statistically significant and P< 0.01 was considered to be very significant.

It is quite obvious that NO was significantly inhibited by pretreatment with M57 in a dose-dependent manner (10, 20, 40, 80 ug/ml) from our result. Also, a similar tendency was observed in NO production at various concentrations of M38 and M56 treated in LPS-stimulated RAW 264.7 cells. In other words, our present study has demonstrated that M38, M56 and M57 have conspicuous inhibitory effect on NO production in LPS-stimulated macrophages, and we can conclude the three compounds could down-regulation LPS-induced iNOS expression. Meanwhile, the finding may explain why M38, M56 and M57 have anti-inflammatory effects in LPS-treated RAW 264.7 cells, and the mechanism of these processes may potentially involve the regulation of directly or indirectly therapeutic targets in NF-κB signal pathway.

## Conclusion

Traditional Chinese Medicine (TCM), a comprehensive and abstruse ethnomedicine, has accumulated thousand years of clinical experiences, however, it lacks the systematical theory and scientific explanation. In this paper, we overcome these drawbacks above and successfully propose a systems pharmacology-based strategy to determine the material bases and the mechanisms of action of traditional Chinese medicinal injection by combining pharmacokinetics (PK) with network pharmacology. Exemplified by Reduning injection, a Chinese medicinal preparation consists of three herbs mainly treating influenza-related diseases, our main findings are:

1. PK modelling, as one of the elements identifying the key properties of a drug, plays an increasingly important role in drug development combining with the druglikeness evaluation. In this work, the systems-based PK models successfully provide 35 candidates (31 molecules and 4 metabolites) for the material basis of Reduning injection. The most common ADME properties are simultaneously and firstly visualized as profiling filter for compound selection in TCM, elucidating the basic and complex trends for multiple properties across various functional groups in a systematic approach.
2. The drug-target and target-disease networks demonstrate the therapeutic polypharmacology of TCM formula, i.e., treating complex diseases by targeting multiple targets with more than one drug in the herbal combination. The herbal medicines with multiple components offer a unique opportunity to explore multiple disease-causing mechanisms simultaneously.
3. The pathway network provides the new way of confronting the influenza with immunomodulatory and anti-inflammatory agents, which may provide clues to making more effective therapeutics against influenza.

As illustrated by Reduning injection, this work demonstrates that the application of our systems pharmacology platform can not only recover the known knowledge of TCM but also provide new findings for uncovering the therapeutic mechanism of herbal injection. The major limitation of this approach is its direct demand for further experimental validation which will be resolved in our next work.

## Electronic supplementary material

Additional file 1: Table S1: The detailed information of all compounds in Reduning Injection. +:compound can overcome the barriers; −:compound cannot overcome the barriers. L: long half-life (half-life value ≥4 h); and S: short half-life (half-life value<4 h). (XLSX 42 KB)

Additional file 2: Table S2: Biological activity (half-life) data and parameters of 169 chemicals in the Drugbank. L: long half-life (half-life value ≥4 h); and S: short half-life (half-life value<4 h). (XLSX 71 KB)

## Abbreviations

TCM: Traditional Chinese medicine
ADME: Absorption, distribution, metabolism, and excretion
PK/PD: Pharmacokinetics-pharmacodynamics
log P: the partition coefficient P
P-gp: P-glycoprotein
PPB: Plasma-protein binding
SVM: Support vector machine
CYP: Cytochromes P450
t_1/2_: Half-life
C-PLS: C-partial least square
TS: Tanimoto similarity
RF: Random forest.

## References

[1] Zhou G, Chen S, Wang Z, Chen Z (2007). Back to the future of oridonin: again, compound from medicinal herb shows potent antileukemia efficacies in vitro and in vivo. Cell Res.

[2] Xu H, Wang Y, Liu N (2009). Safety of an injection with a mixture of extracts from Herba Artemisiae annuae, Fructus Gardeniae and Flos Lonicerae. Pharm World Sci.

[3] Zhang A, Sun H, Wang Z, Sun W, Wang P, Wang X (2010). Metabolomics: towards understanding traditional Chinese medicine. Planta Med.

[4] Quintana-Murci L, Chaix R, Wells RS, Behar DM, Sayar H, Scozzari R, Rengo C, Al-Zahery N, Semino O, Santachiara-Benerecetti AS (2004). Where west meets east: the complex mtDNA landscape of the southwest and central Asian corridor. Am J Hum Genet.

[5] van der Graaf P, Benson N (2011). Systems pharmacology: bridging systems biology and pharmacokinetics-pharmacodynamics (PKPD) in drug discovery and development. Pharm Res.

[6] Zhao S, Iyengar R (2012). Systems pharmacology: network analysis to identify multiscale mechanisms of drug action. Annu Rev Pharmacol Toxicol.

[7] Li P, Chen J, Wang J, Zhou W, Wang X, Li B, Tao W, Wang W, Wang Y, Yang L (2013). Systems pharmacology strategies for drug discovery and combination with applications to CVD. J Ethnopharmacol.

[8] Tao W, Xu X, Wang X, Li B, Wang Y, Li Y, Yang L (2012). Network pharmacology-based prediction of the active ingredients and potential targets of Chinese herbal Radix Curcumae formula for application to cardiovascular disease. J Ethnopharmacol.

[9] Huang C, Zheng C, Li Y, Wang Y, Lu A, Yang L (2013). Systems pharmacology in drug discovery and therapeutic insight for herbal medicines. Brief Bioinform.

[10] Li B, Xu X, Wang X, Yu H, Li X, Tao W, Wang Y, Yang L (2012). A systems biology approach to understanding the mechanisms of action of Chinese herbs for treatment of cardiovascular disease. Int J Mol Sci.

[11] Liu H, Wang J, Zhou W, Wang Y, Yang L (2013). Systems approaches and polypharmacology for drug discovery from herbal medicines: an example using licorice. J Ethnopharmacol.

[12] Yao Y, Zhang X, Wang Z, Zheng C, Li P, Huang C, Tao W, Xiao W, Wang Y, Huang L (2013). Deciphering the combination principles of traditional Chinese medicine from a systems pharmacology perspective based on Ma-Huang decoction. J Ethnopharmacol.

[13] Wang X, Xu X, Tao W, Li Y, Wang Y, Yang L (2012). A systems biology approach to uncovering pharmacological synergy in herbal medicines with applications to cardiovascular disease. Evid Based Complement Altern Med.

[14] Li Y, Wang Z, Bi Y, Ding G, Sheng L, Qin J, Xiao W, Li J, Wang Y, Wang X (2012). The evaluation and implementation of direct analysis in real time quadrupole time-of-flight tandem mass spectrometry for characterization and quantification of geniposide in Re Du Ning injections. Rapid Commun Mass Spectrom.

[15] Li H (2013). Study on the Therapeutical Basis of Composite Herbal Medicines of Reduning Injection.

[16] van de Waterbeemd H, Gifford E (2003). ADMET in silico modelling: towards prediction paradise?. Nat Rev Drug Discov.

[17] Tetko I, Tanchuk V, Villa A (2001). Prediction of n-octanol/water partition coefficients from PHYSPROP database using artificial neural networks and E-state indices. J Chem Inf Comput Sci.

[18] Lipinski C, Lombardo F, Dominy B, Feeney P (2012). Experimental and computational approaches to estimate solubility and permeability in drug discovery and development settings. Adv Drug Deliv Rev.

[19] Sun H (2004). A universal molecular descriptor system for prediction of logP, logS, logBB, and absorption. J Chem Inf Comput Sci.

[20] Jorgensen W, Duffy E (2002). Prediction of drug solubility from structure. Adv Drug Deliv Rev.

[21] Lin J, Yamazaki M (2003). Role of P-glycoprotein in pharmacokinetics: clinical implications. Clin Pharmacokinet.

[22] Wang Y, Li Y, Yang S, Yang L (2005). Classification of substrates and inhibitors of P-glycoprotein using unsupervised machine learning approach. J Chem Inf Model.

[23] Kratochwil N, Huber W, Müller F, Kansy M, Gerber P (2002). Predicting plasma protein binding of drugs: a new approach. Biochem Pharmacol.

[24] Zsila F, Bikadi Z, Malik D, Hari P, Pechan I, Berces A, Hazai E (2011). Evaluation of drug-human serum albumin binding interactions with support vector machine aided online automated docking. Bioinformatics (Oxford, England).

[25] Nelson D, Koymans L, Kamataki T, Stegeman J, Feyereisen R, Waxman D, Waterman M, Gotoh O, Coon M, Estabrook R (1996). P450 superfamily: update on new sequences, gene mapping, accession numbers and nomenclature. Pharmacogenetics.

[26] Saunders KC (2004). Automation and robotics in ADME screening. Drug Discov Today Technol.

[27] Rostkowski M, Spjuth O, Rydberg P (2013). WhichCyp: prediction of cytochromes P450 inhibition. Bioinformatics (Oxford, England).

[28] Manga N, Duffy J, Rowe P, Cronin M (2005). Structure-based methods for the prediction of the dominant P450 enzyme in human drug biotransformation: consideration of CYP3A4, CYP2C9, CYP2D6. SAR QSAR Environ Res.

[29] Madden JC (2010). In Silico Approaches for Predicting ADME Properties. Recent Advances in QSAR Studies.

[30] Kidron H, Del Amo EM, Vellonen KS, Urtti A (2012). Prediction of the vitreal half-life of small molecular drug-like compounds. Pharm Res.

[31] Boulesteix A-L (2004). PLS dimension reduction for classification with microarray data. Stat Appl Genet Mol Biol.

[32] Chung D, Keles S (2010). Sparse partial least squares classification for high dimensional data. Stat Appl Genet Mol Biol.

[33] Knox C, Law V, Jewison T, Liu P, Ly S, Frolkis A, Pon A, Banco K, Mak C, Neveu V (2011). DrugBank 3.0: a comprehensive resource for ‘Omics’ research on drugs. Nucleic Acids Res.

[34] Todeschini R, Consonni V (2008). Handbook of Molecular Descriptors.

[35] Walters W, Murcko M (2002). Prediction of ‘drug-likeness’. Adv Drug Deliv Rev.

[36] Ma C, Wang L, Xie X-Q (2011). GPU accelerated chemical similarity calculation for compound library comparison. J Chem Inf Model.

[37] Rix U, Superti-Furga G (2009). Target profiling of small molecules by chemical proteomics. Nat Chem Biol.

[38] Yu H, Chen J, Xu X, Li Y, Zhao H, Fang Y, Li X, Zhou W, Wang W, Wang Y (2012). A systematic prediction of multiple drug-target interactions from chemical, genomic, and pharmacological data. PLoS One.

[39] Keiser M, Roth B, Armbruster B, Ernsberger P, Irwin J, Shoichet B (2007). Relating protein pharmacology by ligand chemistry. Nat Biotechnol.

[40] Kuhn M, Szklarczyk D, Franceschini A, von Mering C, Jensen LJ, Bork P (2012). STITCH 3: zooming in on protein–chemical interactions. Nucleic Acids Res.

[41] Xu Q, Qu F, Pelkonen O (2012). Network Pharmacology and Traditional Chinese Medicine.

[42] Azuaje FJ, Zhang L, Devaux Y, Wagner DR (2011). Drug-target network in myocardial infarction reveals multiple side effects of unrelated drugs. Sci Rep.

[43] Li S, Zhang B (2013). Traditional Chinese medicine network pharmacology: theory, methodology and application. Chin J Nat Med.

[44] Shannon P, Markiel A, Ozier O, Baliga NS, Wang JT, Ramage D, Amin N, Schwikowski B, Ideker T (2003). Cytoscape: a software environment for integrated models of biomolecular interaction networks. Genome Res.

[45] Archetti F, Lanzeni S, Messina E, Vanneschi L (2006). Genetic Programming for Human Oral Bioavailability of Drugs. Proceedings of the 8th Annual Conference on Genetic and Evolutionary Computation.

[46] Di L, Kerns E (2003). Profiling drug-like properties in discovery research. Curr Opin Chem Biol.

[47] Shang X, Pan H, Li M, Miao X, Ding H (2011). Lonicera japonica Thunb.: ethnopharmacology, phytochemistry and pharmacology of an important traditional Chinese medicine. J Ethnopharmacol.

[48] Chen C, Zhou J, Ji C (2010). Quercetin: a potential drug to reverse multidrug resistance. Life Sci.

[49] Lin Y, Shi R, Wang X, Shen H-M (2008). Luteolin, a flavonoid with potentials for cancer prevention and therapy. Curr Cancer Drug Targets.

[50] Rahden-Staron I, Czeczot H, Szumilo M (2001). Induction of rat liver cytochrome P450 isoenzymes CYP 1A and CYP 2B by different fungicides, nitrofurans, and quercetin. Mutat Res.

[51] Choi J, Piao Y, Kang K (2011). Effects of quercetin on the bioavailability of doxorubicin in rats: role of CYP3A4 and P-gp inhibition by quercetin. Arch Pharm Res.

[52] Ding Y, Zhang T, Tao J, Zhang L, Shi J, Ji G (2013). Potential hepatotoxicity of geniposide, the major iridoid glycoside in dried ripe fruits of Gardenia jasminoides (Zhi-zi). Nat Prod Res.

[53] Tang H, Min G, Ge B, Li Y, Liu X, Jiang S (2008). Evaluation of protective effects of Chi-Zhi-Huang decoction on phase I drug metabolism of liver injured rats by cocktail probe drugs. J Ethnopharmacol.

[54] Chula S, Hang L, Yinying B, Jianning S, Shi R (2012). The effects of notoginsenoside R (1) on the intestinal absorption of geniposide by the everted rat gut sac model. J Ethnopharmacol.

[55] Jang G, Harris R, Lau D (2001). Pharmacokinetics and its role in small molecule drug discovery research. Med Res Rev.

[56] Stoner C, Gifford E, Stankovic C, Lepsy C, Brodfuehrer J, Prasad J, Surendran N (2004). Implementation of an ADME enabling selection and visualization tool for drug discovery. J Pharm Sci.

[57] Boran AD, Iyengar R (2010). Systems approaches to polypharmacology and drug discovery. Curr Opin Drug Discov Devel.

[58] Boots A, Haenen G, Bast A (2008). Health effects of quercetin: from antioxidant to nutraceutical. Eur J Pharmacol.

[59] Spinella M (2002). The importance of pharmacological synergy in psychoactive herbal medicines. Altern Med Rev.

[60] Koshikawa N, Hasegawa S, Nagashima Y, Mitsuhashi K, Tsubota Y, Miyata S, Miyagi Y, Yasumitsu H, Miyazaki K (1998). Expression of trypsin by epithelial cells of various tissues, leukocytes, and neurons in human and mouse. Am J Pathol.

[61] Taubenberger JK, Morens DM (2008). The pathology of influenza virus infections. Annu Rev Pathol.

[62] Qiucheng L (1998). The present state and prospect of the study of syndrome. Zhongguo Zhong Xi Yi Jie He Za Zhi.

[63] Albert D, Zündorf I, Dingermann T, Müller W, Steinhilber D, Werz O (2002). Hyperforin is a dual inhibitor of cyclooxygenase-1 and 5-lipoxygenase. Biochem Pharmacol.

[64] McMillan R (2001). Leukotrienes in respiratory disease. Paediatr Respir Rev.

[65] Wang X, Xu X, Li Y, Li X, Tao W, Li B, Wang Y, Yang L (2012). Systems pharmacology uncovers Janus functions of botanical drugs: activation of host defense system and inhibition of influenza virus replication. Integr Biol (Camb).

[66] Ehrhardt C, Ludwig S (2009). A new player in a deadly game: influenza viruses and the PI3K/Akt signalling pathway. Cell Microbiol.

[67] Fritz J, Ferrero R, Philpott D, Girardin S (2006). Nod-like proteins in immunity, inflammation and disease. Nat Immunol.

[68] Chen G, Shaw M, Kim Y, Nuñez G (2009). NOD-like receptors: role in innate immunity and inflammatory disease. Annu Rev Pathol.

[69] Dinarello C (1996). Biologic basis for interleukin-1 in disease. Blood.

[70] Beg A (2002). Endogenous ligands of Toll-like receptors: implications for regulating inflammatory and immune responses. Trends Immunol.

[71] Akira S, Takeda K, Kaisho T (2001). Toll-like receptors: critical proteins linking innate and acquired immunity. Nat Immunol.

[72] Fedson D (2009). Confronting the next influenza pandemic with anti-inflammatory and immunomodulatory agents: why they are needed and how they might work. Influenza Other Respi Viruses.

[73] Pang IK, Iwasaki A (2011). Inflammasomes as mediators of immunity against influenza virus. Trends Immunol.

[74] Romeo GR, Lee J, Shoelson SE (2012). Metabolic syndrome, insulin resistance, and roles of inflammation–mechanisms and therapeutic targets. Arterioscler Thromb Vasc Biol.

[75] Hsu HY, Wen MH (2002). Lipopolysaccharide-mediated reactive oxygen species and signal transduction in the regulation of interleukin-1 gene expression. J Biol Chem.

[76] Moncada S (1999). Nitric oxide: discovery and impact on clinical medicine. J R Soc Med.

[77] Pasparakis M (2009). Regulation of tissue homeostasis by NF-kappaB signalling: implications for inflammatory diseases. Nat Rev Immunol.

[78] Mancino A, Lawrence T (2010). Nuclear factor-kappaB and tumor-associated macrophages. Clin Cancer Res.

